# Artificial Neural Network Modeling of Mechanical Properties of 3D-Printed Polyamide 12 and Its Fiber-Reinforced Composites

**DOI:** 10.3390/polym17050677

**Published:** 2025-03-03

**Authors:** Catalin Fetecau, Felicia Stan, Doina Boazu

**Affiliations:** 1 Center of Excellence Polymer Processing, Dunarea de Jos University of Galati, Domneasca 47, 800 008 Galati, Romania; catalin.fetecau@ugal.ro; 2 Faculty of Engineering, Dunarea de Jos University of Galati, Domneasca 47, 800 008 Galati, Romania; doina.boazu@ugal.ro

**Keywords:** fusion filament fabrication, polyamide, glass fibers, carbon fibers, mechanical properties, artificial neural networks

## Abstract

Fused filament fabrication (FFF) has recently emerged as a sustainable digital manufacturing technology to fabricate polymer composite parts with complex structures and minimal waste. However, FFF-printed composite parts frequently exhibit heterogeneous structures with low mechanical properties. To manufacture high-end parts with good mechanical properties, advanced predictive tools are required. In this paper, Artificial Neural Network (ANN) models were developed to evaluate the mechanical properties of 3D-printed polyamide 12 (PA) and carbon fiber (CF) and glass fiber (GF) reinforced PA composites. Tensile samples were fabricated by FFF, considering two input parameters, such as printing orientation and infill density, and tested to determine the mechanical properties. Then, single- and multi-target ANN models were trained using the forward propagation Levenberg–Marquardt algorithm. Post-training performance analysis indicated that the ANN models work efficiently and accurately in predicting Young’s modulus and tensile strength of the 3D-printed PA and fiber-reinforced PA composites, with most relative errors being far less than 5%. In terms of mechanical properties, such as Young’s modulus and tensile strength, the 3D-printed composites outperform the unreinforced PA. Printing PA composites with 0° orientation and 100% infill density results in a maximum increase in Young’s modulus (up to 98% for CF/PA and 32% for GF/PA) and tensile strength (up to 36% for CF/PA and 18% for GF/PA) compared to the unreinforced PA. This study underscores the potential of the ANN models to predict the mechanical properties of 3D-printed parts, enhancing the use of 3D-printed PA composite components in structural applications.

## 1. Introduction

Fused filament fabrication (FFF), also known as fused deposition modeling (FDM), is one of the most used Additive Manufacturing (AM) techniques and has recently emerged as a sustainable digital manufacturing technology for producing near-net-shape fiber-reinforced plastic (FRP) composites. It offers several advantages for low- to medium-production volumes over traditional manufacturing processes, such as design freedom, rapid prototyping, and high customization [1,2,3,4,5,6,7]. However, the use of 3D-printed FRP components for structural and load-bearing applications is not always feasible due to their lower mechanical properties as compared to traditional manufacturing processes such as injection molding, with most FFF-printed parts being used as prototypes rather than components for real applications.

In an attempt to overcome the mechanical performance constrains, many studies have investigated different aspects of the process–structure–property of FFF-printed FRP composites [8,9,10,11,12,13,14,15,16,17,18]. However, the effect of process parameters on the mechanical properties of 3D-printed FRP composites is far from being understood and, more importantly, accurately predicted. Apart from the fiber content, the mechanical properties of 3D-printed FRP parts depend greatly on the printing parameters such as infill density and printing orientation or raster angle [5,8,9,12,13,14], as these parameters have a direct impact on the mechanical anisotropy, porosity, inter- and intra-filament voids, and adhesion between layers, which in turn negatively influence the mechanical properties of the FFF-printed parts. Thus, overcoming the weak mechanical properties requires the optimization of printing parameters.

As of today, the optimization of the 3D printing process relies heavily on trial experimental measurements [8,9,10,11,12,13,14,15,16,17]. To assess the effect of process parameters on the mechanical properties and their relationship with printing parameters, in general, a series of wide variety samples are usually 3D-printed as a function of different printing parameters and statistically analyzed to achieve the optimum mechanical properties, resulting in the wastage of materials and time, thereby increasing the overall cost of production [8,9,10,11,12,13,14,15,16,17]. An alternative approach is to develop robust models for a more realistic optimization of the 3D printing process and an accurate prediction of the mechanical behavior of 3D-printed structures.

Machine Learning (ML), as a part of artificial intelligence (AI), has become a subject of intensive research and development in recent years, being applied to identify the interrelation between the input and output variables and to understand various aspects of materials informatics, materials design, and manufacturing [19,20,21,22,23,24]. Integrating ML with AM—both digitized processes—facilitates the transition from time-consuming, trial-and-error experimentation to a more digital transformation of the manufacturing processes, offering nearly instant diagnostics and performance predictions [25,26,27]. Among the various ML algorithms used in AM, such as K-nearest neighbor (K-NN), Naive Bayes, support vector regression (SVR), multiple linear regression, Artificial Neural Network (ANN), Markov decision process (MDP), or Q-learning, ANN emerged as one of the most accurate methods for predicting the mechanical properties without the need for expensive experiments. For example, ANN-based models have been developed to predict mechanical [14,25,26,28,29,30,31,32,33] and physical [34,35] properties, wear [36,37,38] or dimensional accuracy [39,40,41,42] of 3D-printed parts as a function of different process parameters. Due to interpretability and the ability to model nonlinearities and interactions, the developed ANN models show promising results in 3D printing. However, ANN weights and biases are very rarely published, and therefore, the results cannot be reproduced under the FAIR (i.e., Findable, Accessible, Interoperable, and Reusable) principle. On the other hand, given the ongoing development of materials and technologies for 3D printing, ANN modeling remains in its initial stages.

Polyamide 12 (PA 12) is an engineering-grade material used for a wide range of applications, from biomechanical to automotive and aerospace industries, that requires good impact resistance, high tensile strength, and thermal stability [7,8,15,16,17,18,43,44]. However, for specific applications that need a combination of static mechanical properties, such as strength and stiffness, and long-term stability under constant (creep) or cyclic (fatigue) loading, including thermal cycles, reinforcing materials like carbon fibers (CFs) and glass fibers (GFs) are added into the PA matrix to enhance the material’s properties [7,18,43,44]. Carbon fibers can reduce the creep deformation; therefore, if a combination of high mechanical properties and creep resistance is needed, such as in automotive or aerospace applications, CF/PA composites are an excellent candidate. Glass fibers have the ability to absorb energy during impact and enhance thermal stability; therefore, GF/PA composites can be used in applications that require mechanical strength and resistance to thermal degradation or impact [7,18,43].

The scientific literature shows that most of the previous works have focused on the experimental investigation of the effects of printing parameters on the mechanical properties of 3D-printed PA 12 and fiber-reinforced PA 12 composites [8,13,14,15,16,17,18], including the optimization of the FFF process using statistical analysis. However, to the best of our knowledge, as indicated by the literature review, ANN models for predicting the mechanical properties of FFF-printed PA 12 or fiber-reinforced PA 12 composites are not readily available.

This study explores the effectiveness of the ANNs to predict the mechanical properties of FFF-printed polyamide 12 and its CF- and GF-reinforced composites. Firstly, tensile samples were FFF-printed and tested to determine the mechanical properties as a function of printing orientation and infill density. To obtain a preliminary assessment of the effect of printing parameters on the mechanical properties, the experimental data were analyzed using analysis of variance. Then, ANN models for tensile strength and Young’s modulus were trained with the Levenberg–Marquardt algorithm and tested with very high accuracy.

## 2. Materials and Methods

### 2.1. Materials

Polyamide 12 (PA), carbon fibers (CF), and glass fibers (GF) were used as matrix and fiber-reinforcing materials. PA (Nylforce^3^, melting point 178 °C, density 1.03 g/cm^3^), CF/PA (Nylforce Carbon fiber 20 wt%, melting point 180 °C, density 1.0 g/cm^3^) and GF/PA (Nylforce Glass fiber 18 wt%, melting point 180 °C, density 1.07 g/cm^3^) filaments were supplied by Fiber Force (Treviso, Italy) in a standard 1.75 ± 0.05 mm diameter form [45]. Table 1 shows the filament specifications according to the manufacturer [45].

### 2.2. Printing Procedure and Design of Experiments

**Figure 1:**
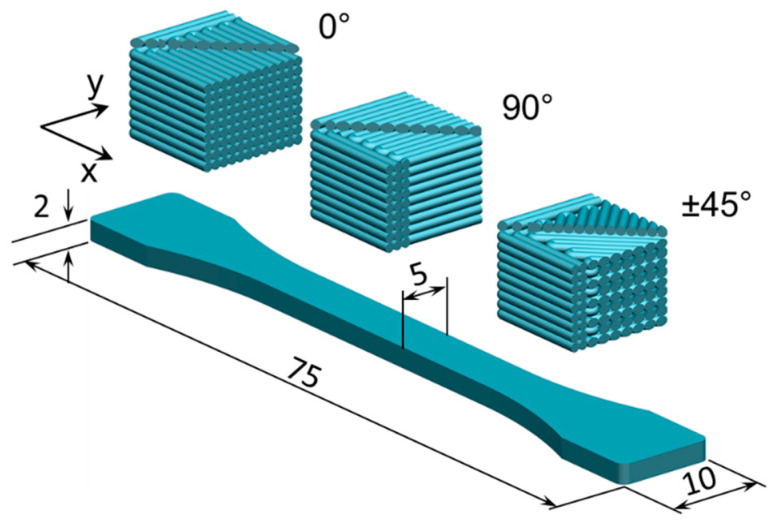
Geometry of the 3D-printed samples and printing direction (all dimensions in mm).

Samples were 3D-printed according to ISO 527-2 type 1BA [46] as illustrated in Figure 1. All samples were fabricated based on the FFF principle using a commercial printer (Prusa i3MK3S, Prusa Research, Prague, Czech Republic) equipped with a nozzle diameter of 0.4 mm. The slicing and FFF processes were controlled using the Prusa slicer (Prusa Research, Prague, Czech Republic).

The orientation of the 3D-printed beads and fibers with respect to the applied load significantly affects the mechanical properties of the 3D-printed parts [47,48,49,50]. The volume fraction can also influence the mechanical properties of the 3D-printed parts, with higher volume fractions resulting in higher strength [47,48,49,50]. Therefore, a full-factorial design of experiment (DoE) was employed to investigate the effect of the infill density (i.e., volume fraction) and printing orientation (referring to the direction of raster with respect to the loading direction) on the mechanical properties, as presented in Table 2.

The remaining printing parameters (e.g., extruder temperature, bed temperature, printing speed, and layer height) were optimized based on several trials, starting from the recommendations provided by the filament supplier [45]. Since the PA12 matrix has a narrow printing temperature window, the effects of the nozzle and bed temperatures were not considered as variables. The nozzle temperature was adjusted to 260 °C and the bed temperature to 100 °C. The printing speed was set to 40 mm/s, the flow to 100%, and the layer height to 0.2 mm. To prevent ambient moisture absorption, the 3D-printed samples were stored in an airtight container after printing until the tensile experiment.

### 2.3. Tensile Testing

Tensile tests were conducted using a universal testing machine (Testometric 350-5AT, Testometric, Rochdale, UK), with a force and displacement measurement accuracy of 0.025 N and 0.001 mm, respectively. The samples were fixed in the grips at a gauge length of 58 mm, and the strain values were determined by using the machine crosshead motion. For each material and experimental run in Table 1, at least seven specimens were tested at a 5 mm/min crosshead speed. The mechanical properties, such as Young’s modulus (E), tensile strength (TS), stress at break (SB), and strain at break (EB), were directly extracted from the engineering stress–strain curves.

### 2.4. SEM Analysis

Cryo-fractured surfaces of the 3D-printed samples were observed in the gauge area using a scanning electron microscopy (SEM, Quanta 200, FEI, Hillsboro, OR, USA), at an accelerating rate of 25 kV. The samples were coated with a thin layer of gold before SEM analysis.

### 2.5. Statistical Analysis

All data were obtained from independent experiments with replication and expressed as mean ± standard deviation (DS). Statistical methods such as Grubb’s test for outliers and the Anderson Darling test for normality were used to analyze the experimental data before performing the analysis of variance (ANOVA) with Tukey’s pairwise comparison, and regression analysis. Furthermore, the two-sample *t*-test was employed to test the significance of the difference between the experimental and ANN prediction data. The statistical analysis was performed with Minitab statistical software (Minitab 19 statistical software (Minitab LLC, State College, PA, USA) with a *p*-value < 0.05 as statistically significant [51].

### 2.6. Artificial Neural Network (ANN) Topology and Training

**Figure 2:**
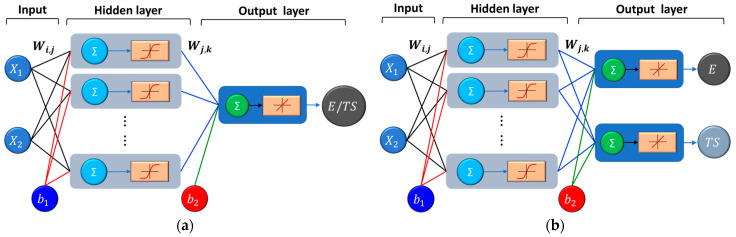
Configuration of the feed-forward neural network: (**a**) one output (Young’s modulus or tensile strength); (**b**) two outputs (Young’s modulus and tensile strength).

To develop an ANN model that gives the best prediction, several aspects must be considered, including the network topology, the number of layers, the number of neurons in the hidden layers, the transfer function, etc., [31,52]. In this study, a multilayer feed-forward ANN model has been developed, as shown in Figure 2. The ANN model considered in this study consists of one input layer with two variables (e.g., infill density and printing orientation), a hidden layer of sigmoidal neurons, and an output layer with one or two variables (e.g., Young’s and/or tensile strength) of linear neurons, as shown in Figure 2.

A sigmoid transfer function and a linear transfer function were used as the activation functions for the hidden layer and output layer, respectively. The sigmoid function (SIG) and linear transfer function (PURELIN) are given by the following in (1) and (2), respectively [52,53]:

(1) $$SIG=y=\frac{1}{1+exp(-x)}$$

(2) PURELIN=y=x

Using the notations in Equations (1) and (2), the network output can be given by Equation (3)

(3) $$y_{i}=PURELIN\left(W_{0}\times \left[SIG\left(W_{i}\times x_{k}+b_{1}\right)\right]+b_{2}\right)$$

Each input ($x_{i}$) is assigned with an appropriate weighting factor ($W_{i}$). The sum of the weighted inputs and the bias produces the input for the transfer function, which will generate an output [52,53]. The output layer computes the weighted sum of the signals provided by the hidden layer, and the associated coefficients are grouped into matrices $W_{0}$ and $b_{2}$.

In this study, the number of neurons in the hidden layer was iteratively determined. To train the network and identify the optimum number of neurons on the hidden layer, two scenarios were considered, as shown in Figure 2. In the first scenario (Figure 2a), a single target ANN model was developed, in which the output layer has only one output, and the ANN was trained separately for the elastic modulus and tensile strength. The second scenario (Figure 2b) involves a multi-target ANN model, and the ANN was simultaneously trained for Young’s modulus and tensile strength.

To minimize the differences between the target (experimental data) and the output (the response of the ANN generated by a weight-adjusting process), the Levenberg–Marquardt forward optimization algorithm was used [53]. All calculations were carried out using MATLAB mathematical software (version R2018a) with the ANN toolbox [53].

The performance of the ANN model can be evaluated using different statistical performance indicators, such as the mean square error (MSE), mean absolute error (MAE), coefficient of determination ($R^{2}$), linear regression coefficient (R), and relative error (ε) [52,53,54,55]. In this study, the MSE, $R^{2}$, and ε, were used to evaluate the performance of different networks based on which an optimum network is selected [52,53,54,55]:

(4) $$MSE=\frac{\sum_{i=1}^{N}{\left(y_{ANN,i}-y_{EXP,i}\right)}^{2}}{N},$$

(5) $$R^{2}=1-\frac{\sum_{i=1}^{N}{\left({y_{EXP,i}-y}_{NN,i}\right)}^{2}}{\sum_{i=1}^{N}{\left({y_{EXP,i}-\bar{y}}_{EXP,i}\right)}^{2}};\;R=\surd {(R}^{2}),$$

(6) $$\varepsilon =\frac{y_{ANN}-y_{EXP}}{y_{EXP}}\times 100\%$$

where $y_{ANN}$ is the output or the response of the ANN for the given input, $y_{EXP}$ is the target value of the corresponding input (i.e., the measured experimental value of the variable), and N is the number of observations in the training or testing data sets.

Aiming to optimize prediction accuracy while minimizing the number of experiments, in this study, the DoE was combined with ANN modeling [56]. Therefore, the ANN model was trained using a 3^2^ full factorial design (two factors, each at three levels) considering the average of five individual measurements. Table 3 presents the input and the output variables for the ANN modeling.

The run order and the values of the input parameters for the ANN modeling are given in Table 2. After training, a separate data set was used to evaluate the ANN performance. To generate the material data set for testing, for each experimental line in the full factorial design, one value was randomly extracted from the five experimental values.

## 3. Results and Discussion

### 3.1. Fracture Morphology of FFF-Printed Samples

In the FFF process, the fibers are preferentially aligned along the deposition path due to high shear rates between the melt and the nozzle wall [47,48,49,50], and the orientation is preserved due to fast cooling rates [8,17,57]. While fiber pull-out, fiber rupture, and fiber-matrix interfacial debonding are the dominating failure modes in FFF-printed glass and carbon fiber-reinforced composites [8,10,11], several studies have also reported the presence of inter- and intra-layer voids (porosity), including the formation of void around the fibers [9,10,16,47,58].

**Figure 3:**
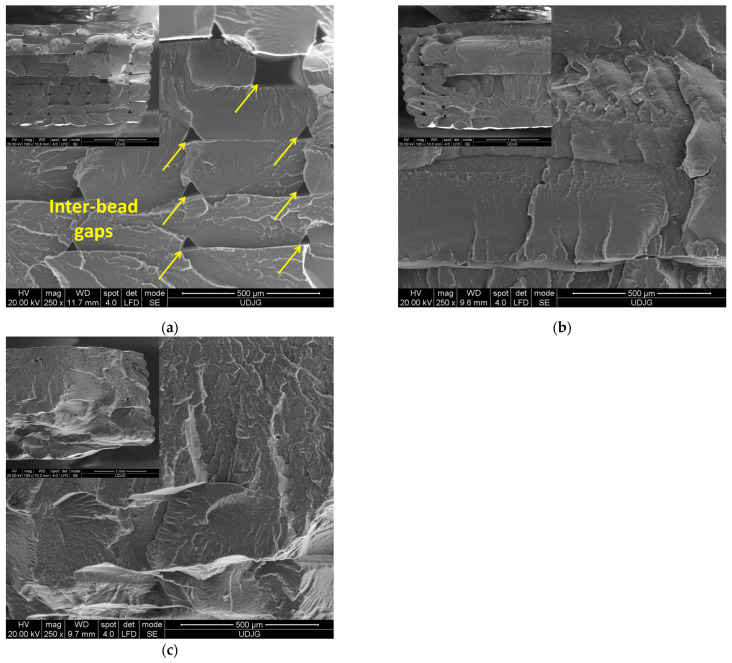
SEM images of the fractured surface of the FFF-printed PA sample with (**a**) 0°, (**b**) 90°, and (**c**) ±45° printing orientation.

The fractured surfaces of the neat PA tensile samples printed with 0°, 90°, and ±45° orientations are shown in Figure 3. The SEM micrographs reveal a clear distinction between the 0°, 90°, and ±45° printing orientations. The fractured surface of the PA samples printed with a 0° orientation (Figure 3a) shows clearly visible individual 3D-printed layers (beads) oriented along the printing direction and typical regular inter-bead triangular gaps (voids).

The shape of the 3D-printed layers and the material flow variation during the 3D-printing process cause the formation of pores inside the 3D-printed parts [59,60]. On the other hand, the SEM micrographs of the PA samples printed with 90° and ±45° orientation show only gaps between the perimeters, while it is difficult to distinguish the individual beads, as shown in Figure 3b,c. Moreover, at a higher magnification, the micrographs clearly show very insignificant interlayer voids and a surface like that of a homogenous material.

**Figure 4:**
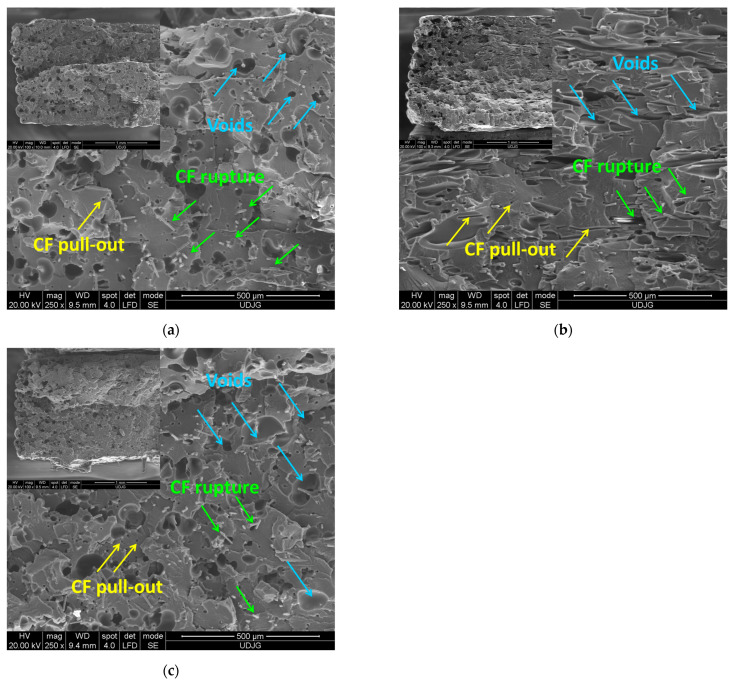
SEM images of the fractured surface of the FFF-printed CF/PA sample with (**a**) 0°, (**b**) 90°, and (**c**) ±45° printing orientation.

The fractured surfaces of the CF/PA samples printed with 0°, 90°, and ±45° orientations are shown in Figure 4. The FFF-printed CF/PA samples display a porous-like structure with high number of voids, including dimples (pores) in the polymer matrix, formed around the CFs. Although the FFF process involves the layer-to-layer deposition, the individual beads are difficult to distinguish on the fractured surface of the CF/PA composite. Close observation of the SEM images clearly displays fiber pull-outs and significant fiber rupture, with carbon fibers being highly aligned along the bead deposition path (Figure 4b). Moreover, holes are also observed on the fractured surfaces of the CF/PA samples, indicating a good adhesion between the fibers and the matrix.

**Figure 5:**
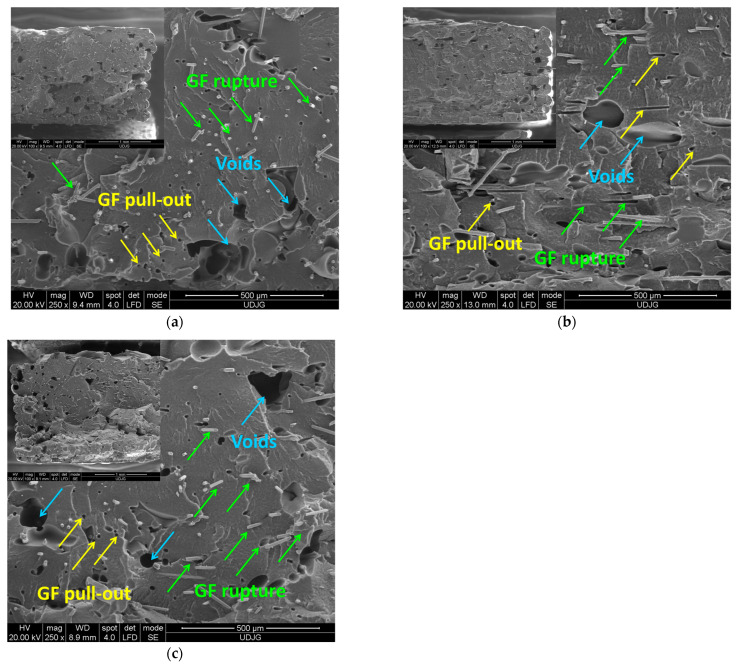
SEM images of the fractured surface of the FFF-printed GF/PA sample with (**a**) 0°, (**b**) 90°, and (**c**) ±45° printing orientation.

The surface morphology of the FFF-printed GF/PA samples is presented in Figure 5. Similar to CF/PA composites, the individual 3D-printed layers cannot be distinguished on the fractured surface of the specimen manufactured from GF/PA composite. However, Figure 5 shows that the porosity of glass fiber-reinforced PA composite is reduced as compared with the carbon fiber-reinforced PA composites. The FFF-printed GF/PA composites display large voids and significant fiber breakage, indicating that adhesion between the fibers and the PA matrix was very good. Also, the SEM images show that the glass fibers that are aligned along the printing directions (see, Figure 5b).

For both FFF-printed CF/PA and GF/PA composites, the fracture surface indicates the absence of inter-layer voids or delamination and the formation of different types of voids (e.g., dimples around the fibers, holes, mainly due to the fiber pull-out, and several large voids (gaps). Similar observations were reported in the literature for 3D-printed carbon fiber and glass fiber-reinforced polymers [10,16,17,58,60,61,62]. The voids around the fibers may be attributed to the severe swirling generated by the fountain flow [16]. On the other hand, the porosity of 3D-printed parts with short carbon fiber reinforced PA12 depends on the fiber content, the printing parameters such as the nozzle temperature, and also on the printed geometry, as reported by several studied [59,60,61,62].

### 3.2. Stress–Strain Behavior of FFF-Printed Samples

**Figure 6:**
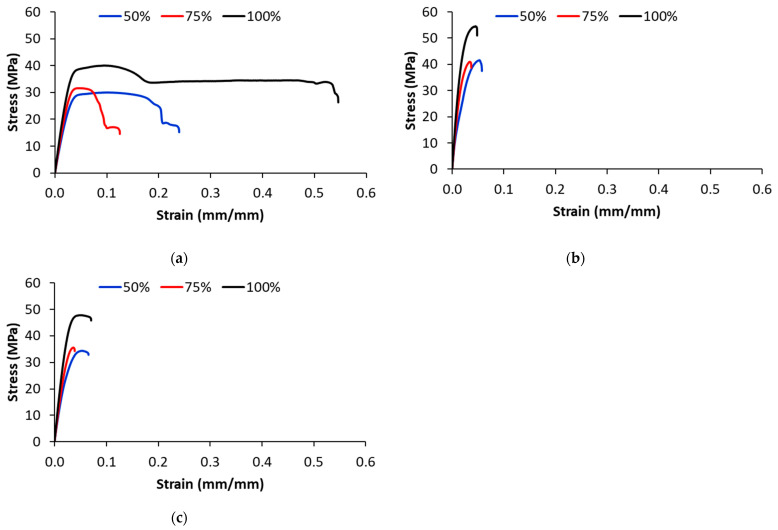
Representative stress–strain curves for 3D-printed samples with 0° printing orientation: (**a**) PA; (**b**) CF/PA; (**c**) GF/PA.

Figure 6 shows the effect of the infill density on the engineering stress–strain tensile curves of the PA-based samples printed with a 0° printing orientation. For all FFF-printed materials, the stress increased with increasing infill density. The 3D-printed unreinforced PA parts show a ductile behavior that is significantly influenced by the infill density (Figure 6a).

All the FFF-printed composites experience brittle behavior with little to no yielding (Figure 6b,c). Considering the results for 100% infill density, the PA parts exhibited a longitudinal tensile strength of 40 MPa, whereas the GF/PA and CF/PA parts displayed an ultimate strength of 45 MPa and 50 MPa, respectively.

**Figure 7:**
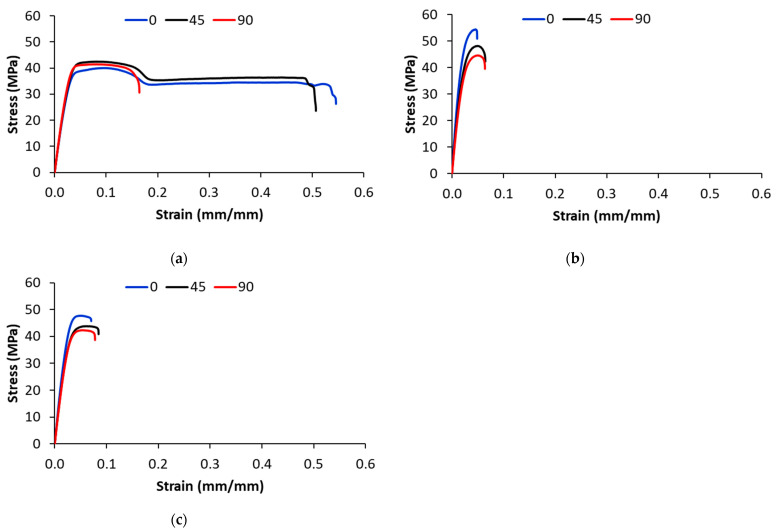
Effect of printing orientation on the stress–strain curves of 3D-printed (**a**) PA, (**b**) CF/PA and (**c**) GF/PA (100% infill density).

Figure 7 shows the effect of printing orientation on the stress–strain curves of 3D-printed samples with 100% infill density. For the 3D-printed PA samples, the effect of the printing direction on the load-bearing capacity is marginal (Figure 7a). However, composite samples printed with 0° orientation have higher a tensile strength than ones printed with ±45° and 90° orientation. As the test is performed in the longitudinal direction, the fibers in the 0°-oriented beads are parallel to the applied load, increasing the load-bearing capacity of the 3D-printed composite samples. As the printing angle changes to ±45° and 90°, the load-bearing capacity of the fibers reduces (Figure 7b,c).

As can be observed from the stress–strain curves of the 3D-printed PA samples in Figure 7a, the elongation at break is affected by the printing orientation; particularly, the samples printed with a 90° printing orientation that is perpendicular to the applied load have the lowest elongation at break (e.g., the elongation at break decreased by 68% as compared with ±45° and 0° orientation). For 3D-printed CF/PA and GF/PA samples, the effect of the printing orientation on the elongation at break is marginal. The 3D-printed composite samples exhibit brittle behavior with no plastic deformation before fracture.

**Figure 8:**
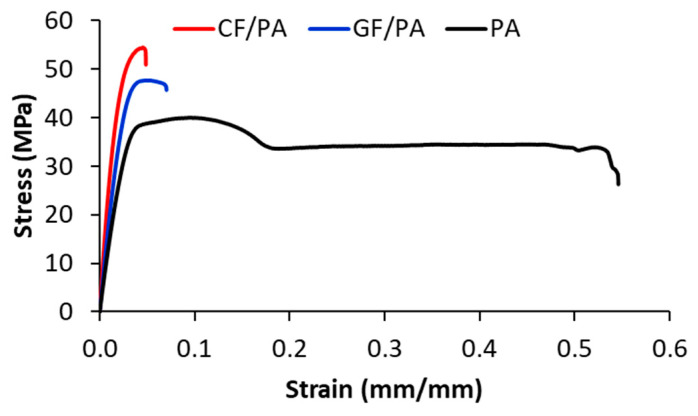
Effect of fillers on the stress–strain curves of samples 3D-printed with 0° printing orientation and 100% infill density.

The effect of fillers on the mechanical behavior of the samples printed with 0° orientation and 100% infill density is illustrated in Figure 8. The maximum stress increases with the addition of fibers, while the elongation is negatively affected by the addition of fibers. The fibers inside the 0°-oriented beads provide resistance to fracture, and therefore the load-bearing capacity increased with the addition of glass and carbon fibers [48,49,50]. The elongation at break drops sharply in the 3D-printed composites due to the increase in defect density (voids), and this is supported by the SEM analysis. On the other hand, the higher elongation at break of the glass fiber-reinforced composites indicates that the glass fibers are more flexible than carbon fibers [48,49,50]. Regarding the fracture mechanism, the 3D-printed PA samples exhibit ductile fracture with necking, while the PA-based composite samples exhibit brittle fracture after reaching the maximum load-bearing capacity.

### 3.3. Mechanical Properties

**Figure 9:**
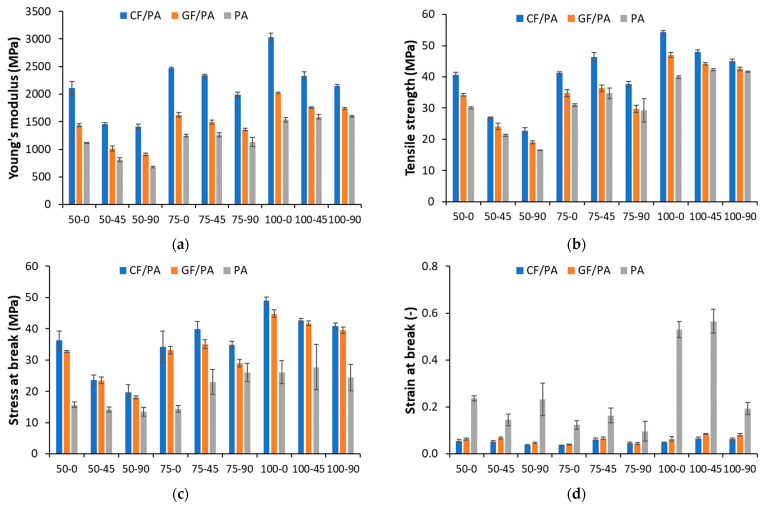
Mechanical properties of 3D-printed PA and PA-based composites (**a**) Young’s modulus, (**b**) tensile strength, (**c**) stress at break, and (**d**) strain at break.

The mechanical properties (e.g., Young’s modulus, tensile strength, stress at break, and strain at break) as a function of infill density and printing orientation are plotted in Figure 9. The mechanical properties of the 3D-printed CF/PA and GF/PA composites are very repeatable. For the 3D-printed PA samples, the initial elastic response and tensile strength are very repeatable, but the failure response shows more variation than the composite samples, both in stress and elongation at break.

Considering the results in Figure 9, it can be observed that the 3D-printed PA-based composites have better mechanical properties compared to the 3D-printed PA, except for the elongation at break. The addition of CF and GF has exhibited a significant improvement in tensile strength and modulus, which can be attributed to the high stiffness of CF and GF when compared to the PA matrix. On the other hand, the stiffness, tensile strength, and stress at break of 3D-printed CF/PA samples are clearly higher than those of GF/PA and PA samples, regardless of the printing parameters. This behavior can be attributed to the higher stiffness of carbon fibers as compared with the glass fibers.

According to the experimental data in Figure 9, a tensile strength of 40 MPa and an elastic modulus of 1537 MPa are obtained for PA samples printed with 0° printing orientation and 100% infill density. The addition of GF and CF leads to an 18% and 36% increase in tensile strength and a 32% and 98% increase in the elastic modulus. While CFs and GFs reinforcement increased the Young’s modulus and tensile strength, they have significantly decreased the elongation at break as compared with the neat PA. The FFF-printed composite samples have comparable behavior with less than 10% elongation at break, showing a brittle failure, whereas, despite large variations in elongation at break, the neat PA samples exhibit ductile failure with elongation at break in the range of 15–60%, depending on the printing orientation and infill density.

**Figure 10:**
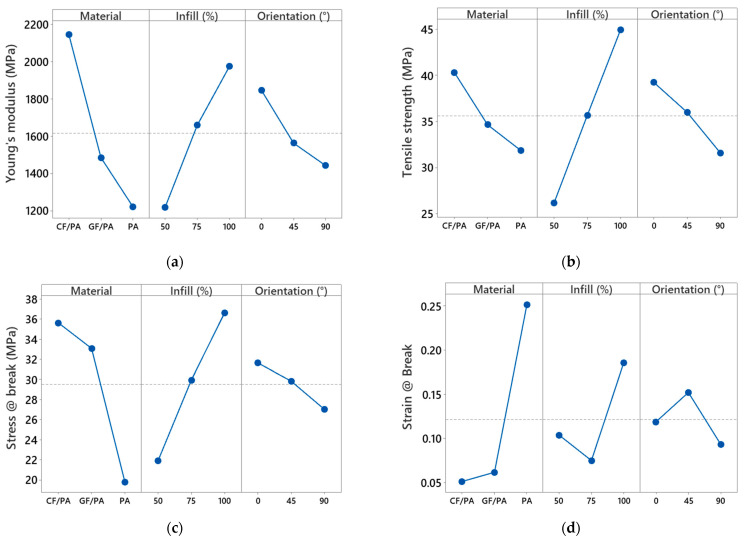
Main effects plot for 3D-printed PA-based samples: (**a**) Young’s modulus, (**b**) tensile strength, (**c**) stress at break, and (**d**) strain at break.

The effects of printing parameters and fillers on the mechanical properties are illustrated in the main effects plots, as shown in Figure 10. The mechanical properties are sensitive to both printing orientation and infill density.

Specifically, the Young’s modulus and tensile strength increased with increasing infill density. Samples printed with 90° orientation display the lowest mechanical properties (Young’s modulus, tensile strength, and stress at break), as the applied load is perpendicular to the 3D-printed layers. The addition of GF and CF significantly improved the mechanical properties of the PA matrix. 3D-printed CF/PA composites exhibit the highest mechanical properties as compared with GF/PA and PA.

Statistical analysis in the form of ANOVA was performed to assess the influence of the main factors (e.g., filler, printing orientation, and infill density) on the response variables (e.g., Young’s, tensile strength, stress at break, and strain at break). Table 4 summarizes the results for *F*-value and the corresponding *p*-value from the ANOVA Table A1, Table A2, Table A3 and Table A4 (Appendix A). For the analyzed response variables, the significant factors are the main factors as well as all the two-way interactions and three-way interactions (*p*-value < 0.05). As observed, the main factors are by and large more significant than the interaction effects as described by their high *F*-value.

For each 3D-printed material, predictive regression models were developed for the Young’s modulus and tensile strength as a function of infill density and printing orientation, as presented in Table 5. The developed regression models can estimate the mechanical properties with a very high accuracy (*R* values of 0.93–0.99).

### 3.4. ANN Models for Young’s and Tensile Strength

#### 3.4.1. ANN with One Output

To determine the optimal ANN architecture, various ANN configurations were tested, considering different numbers of neurons in the hidden layer (Figure 2a). The *R* and the *MSE* were used to identify the best network (i.e., the optimal number of neurons in the hidden layer) with the least error during the training stage. Table 6 presents the *R* and *MSE* values as a function of the number of neurons in the hidden layer. The lowest *MSE* value and highest *R* value indicate that the model predicts the mechanical properties with the best accuracy. The best predictions for both Young’s modulus and tensile strength are obtained by the ANN with three neurons in the hidden layer; therefore, the best-performing ANN model is 2-3-1. Table 7 and Table 8 summarize the computed statistical parameters (weights and biases) for the optimum ANN models.

**Figure 11:**
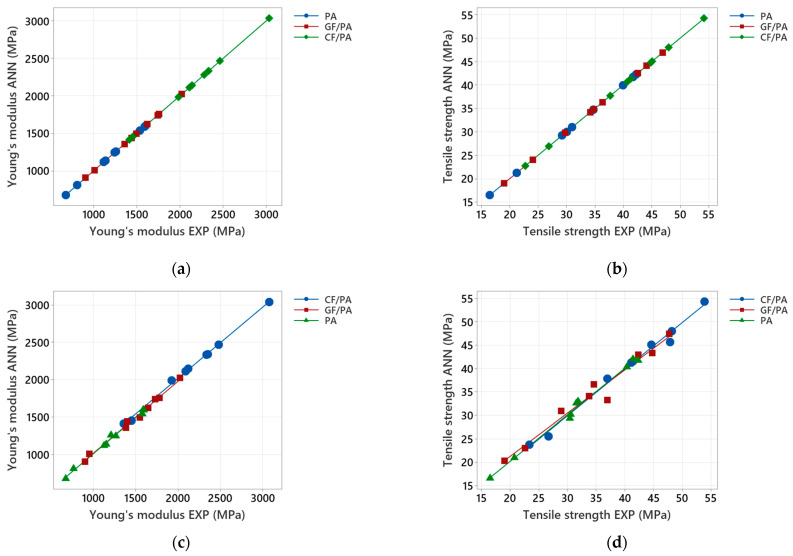
Comparison of experimental Young’s modulus and tensile strength with the predicted values for the optimized ANN model with one output: (**a**,**b**) Training results; (**c**,**d**) Testing results.

The performance of the ANN models is illustrated in Figure 11, where the ANN outputs are plotted against the targets for both training and testing data sets. For the training stage, an almost perfect fit is indicated in Figure 11a,b, and the Young’s modulus and tensile strength outputs for all the investigated 3D-printed materials track the targets very well (e.g., the *R* values are 1.0).

Regarding the testing performance of the ANN models, Figure 11c,d indicate that, although the network outputs are slightly more scattered, particularly for the tensile strength, both output variables (Young’s modulus and tensile strength) compare very well. The testing network outputs versus the targets, along with relative error, are presented in Table A5, Table A6 and Table A7 (Appendix B). As shown in these tables, the ANN models (three neurons in the hidden layer) make very high-quality predictions, indicating that most of the relative errors are less than 5%, with an average relative error of less than 2% for both mechanical properties. The Pearson correlation coefficient in Table 9 indicates a strong statistically significant correlation between the experimental and ANN-predicted values for the testing data sets (*p*-value < 0.0005). Therefore, it can be concluded that the ANN model with one output demonstrates the capability to model the mechanical properties of 3D-printed PA and PA-based composites.

#### 3.4.2. ANN with Two Outputs

In the second scenario, the ANN was simultaneously trained considering two outputs, namely Young’s modulus and tensile strength, as explained in Section 2 and shown in Figure 2b. Table 10 presents the variation of *R* and *MSE* with the number of neurons in the hidden layer. Based on the value of *R* (the highest) and *MSE* (the smallest), the optimum architecture of the ANN was identified as follows: for the CF/PA, the best prediction is achieved with four neurons in the hidden layer (2-4-2), while for the GF/PA and PA, the best prediction is achieved with three neurons in the hidden layer (2-3-2). Table 11 summarizes the final weights and bias for the ANN model with two outputs.

**Figure 12:**
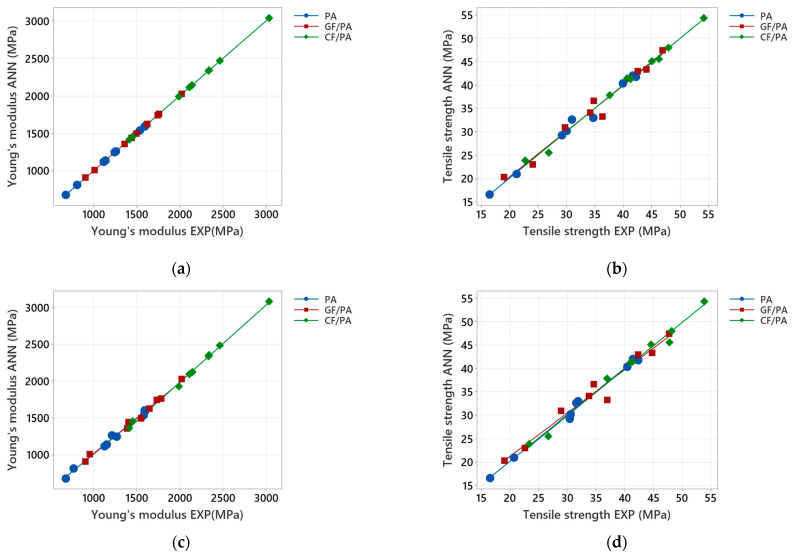
Comparison of experimental Young’s modulus and tensile strength with the predicted values for the optimized ANN model with two outputs: (**a**,**b**) Training results; (**c**,**d**) Testing results.

The performance of the trained ANN models with two outputs is illustrated in Figure 12, where the outputs are plotted against the targets for both training and testing data sets. As shown in Figure 12a,b, the ANN models are capable of simultaneously learning the effects of printing orientation and infill density on the mechanical properties of the 3D-printed materials (Young’s modulus and tensile strength).

For Young’s modulus, Figure 12a shows that all the targets and ANN outputs are located on the diagonal line, indicating that the accuracy of the training process is very high. As presented in Table 12, a perfect correlation between targets and outputs is obtained (e.g., R equals 1).

However, for tensile strength, particularly for the GF/PA composites, the training data are more scattered, as indicated in Figure 12b, but still, the ANN outputs track the targets reasonably well, as the *R* value is close to 1 (Table 12). Moreover, all the Pearson’s correlation coefficients are statistically significant, with *p*-value < 0.0005 (Table 12). It can be concluded that the ANN model works efficiently and accurately in predicting the Young’s modulus and tensile strength of the 3D-printed PA and PA-based composites.

To evaluate the generalization ability of the trained networks in predicting mechanical properties, the outputs and the targets in the testing data set are presented in Figure 12c,d, respectively. Although Figure 12d shows a slightly more scattered pattern, all the points still fall within a reasonably close range of the diagonal. For the sake of comparison, the experimental and ANN-predicted values, along with the relative errors at the testing stage, are listed in Table A8, Table A9 and Table A10 (Appendix C). For the 3D-printed PA and CF/PA composites, the ANN model provides very high-quality predictions, with all relative errors being less than 5% (Table A8 and Table A9), and a mean average relative error below 2%. For the 3D-printed GF/PA composite, while the ANN model yields high predictions for Young’s modulus with an average relative error under 2%, it achieves medium to high-quality predictions for tensile strength in four out of nine cases, showing relative errors below 10% (Table A10). However, most relative errors are less than 5%, and the average relative error is less than 4%. The Pearson correlation coefficient in Table 12 suggests a strong positive correlation between the experimental and ANN-predicted values for both Young’s modulus and tensile strength. Pearson’s correlation coefficient is statistically significant with a *p*-value < 0.0005, indicating that the optimized ANN models are very accurate in predicting the Young’s modulus and tensile strength of the 3D-printed PA and PA-based composites.

#### 3.4.3. Comparison of the ANN Models

As shown in the previous sub-sections, the performance of the ANN models was measured by the mean square error, correlation coefficient, and relative errors on the training and testing data sets, as well as by performing regression analysis along with the Pearson’s correlation coefficient between the network responses and the corresponding targets.

After the ANN models were trained and tested, the network performance was further assessed considering the entire data set. Therefore, the entire data set (training and testing) was put through the network and analyzed using the linear regression between the network outputs and the corresponding targets. Table 13 shows the Pearson’s correlation along with the corresponding *p*-value.

As can be seen in Table 13, while both trained networks (with one and two outputs) are, overall, capable of performing accurately, the ANN model with one output performs better in predicting the tensile strength for 3D-printed composites. On the other hand, the trained ANN models tend to be more robust for predicting the Young’s modulus. This could be explained by the fact that some 3D-printed PA parts have large standard deviations for tensile strength.

The accuracy of the prediction from the trained ANN models was further addressed from a statistical perspective by using the two-sample *t*-test to compare ANN model predictions (outputs) with experimentally measured mechanical properties (targets), at a significance level of 0.05. Based on the results presented in Table 14, it can be concluded that the difference between the predicted and experimentally measured mechanical properties is not statistically significant (*p*-value > 0.05).

#### 3.4.4. Validation of the ANN Models

**Figure 13:**
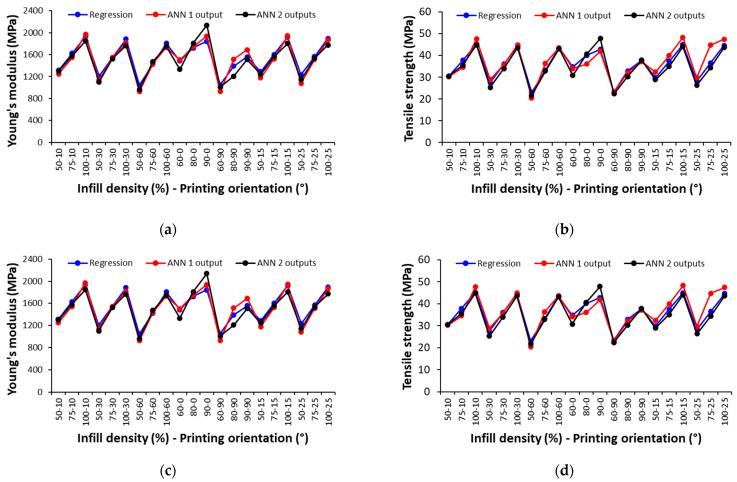

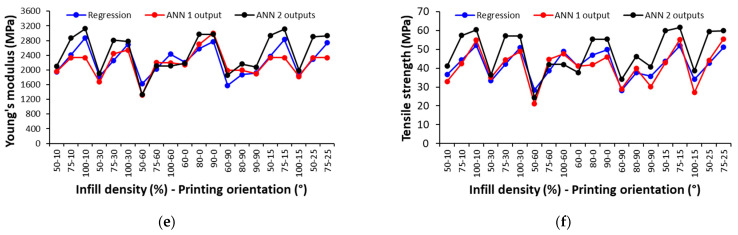
Comparison between the predicted outcomes using the regression and ANN models for 3D-printed: (**a**,**b**) PA, (**c**,**d**) GF/PA; and (**e**,**f**) CF/PA samples.

To assess the predictive performance of the developed ANN models for unseen data sets and their generalization capability, in the absence of additional experimental data, the outcomes (e.g., Young’s modulus and tensile strength) were predicted using both the ANN and regression models for various combinations of unseen input parameters. Figure 13 compares the outcomes (e.g., Young’s modulus and tensile strength) for the ANN models and regression models calculated for each 3D-printed material.

For 3D-printed PA and GF/PA composites, the developed ANN models estimate the mechanical properties closely to the regression results, with most of the relative errors well below 10%. However, for the 3D-printed CF/PA composites, the ANN models perform slightly less accurately, particularly the ANN model with two outputs predicted mechanical properties higher than those predicted by the regression model, suggesting potential challenges regarding the generalization capability of the ANN model. It should be noted that the correlation coefficient of the regression model for the CF/PA composites is 0.93–0.94 (Table 5), indicating that, in addition to the infill density and printing orientation, the mechanical behavior of the 3D-printed CF/PA composites may be influenced by other factors.

Even though the ANN models for CF/PA composites perform less accurately with correlation coefficients below 0.9 (Table 15), the developed ANN models are generally robust in predicting the mechanical properties across unseen data sets, with correlation coefficients that are statistically significant, as indicated in Table 15 (all *p*-value < 0.005).

## 4. Conclusions

This paper reported on the neural network modeling of the mechanical properties of 3D-printed polyamide 12 and its CF- and GF-reinforced composites. Samples were fabricated by FFF using a 3^2^ full factorial DoE, considering two input parameters, such as printing orientation and infill density. All FFF-printed samples were subjected to tensile testing, and the mechanical properties were statistically analyzed using the two-way ANOVA, and regression models were developed for Young’s modulus and tensile strength for each 3D-printed material. Two types of ANN models with a forward propagation Levenberg–Marquardt algorithm were trained and tested for the Young’s modulus and ultimate tensile strength. The first model is a single-target ANN model for predicting one mechanical property (either Young’s modulus or tensile strength). The second model is a multi-target ANN model for simultaneously predicting two mechanical properties (e.g., tensile strength and Young’s modulus). The performance of the ANN models was assessed on the training and testing data sets based on several statistical indicators (e.g., the mean square error, relative error, average relative error, and correlation coefficient). The comparisons between the target and output mechanical properties demonstrate that the accuracy of the training process is very high, and the ANN models can predict the mechanical properties accurately, with relative errors generally lower than 5%. A good agreement was found between the ANN and regression predictions for different combinations of the unseen data sets, suggesting the capacity of the developed ANN model to capture the patterns in the Young’s modulus and tensile strength.

In terms of mechanical properties, such as Young’s modulus and tensile strength, the 3D-printed composites outperform the unreinforced PA. Printing PA composites with 0° orientation and 100% infill density results in a maximum increase in Young’s modulus (up to 98% for CF/PA and 32% for GF/PA) and tensile strength (up to 36% for CF/PA and 18% for GF/PA) compared to the unreinforced PA.

This study demonstrates the advantage of coupling DoE with ANNs to predict the mechanical properties of 3D-printed composites, enhancing their use in structural applications. Future work will address the ANN modeling by considering additional 3D printing parameters, including porosity, anisotropy, and filler content. Furthermore, various training algorithms will be examined to enhance the robustness of the trained ANN models.

## Figures and Tables

**Table 1:** Filament printing specifications [45].

Parameter	PA	GF/PA	CF/PA
Printing temperature (°C)	240–250	250–265	250–265
Bed temperature (°C)	70	60–70	60–70
Print speed (mm/s)	30–50	40	40

**Table 2:** Printing parameters and levels.

Run	Infill Density (%)	Printing Orientation (°)
1	50	0
2	50	±45
3	50	90
4	75	0
5	75	±45
6	75	90
7	100	0
8	100	±45
9	100	90

**Table 3:** Characteristics of input and output variables of the ANN model.

Experimental Variables	Value
Input	
$\mathrm{Infill}\;\mathrm{density},\;X_{1}$ (%)	50, 75, 100
$\mathrm{Printing}\;\mathrm{orientation},\;X_{2}$ (°)	0, 90, ±45
Output	
Young modulus, E (MPa)	
Tensile strength, TS (MPa)	

**Table 4:** ANOVA on average mechanical properties (p−value<0.05).

Source	df	Young Modulus		Tensile Strength		Stress at Break		Strain at Break	
		F-Value	*p*-Value	F-Value	*p*-Value	F-Value	*p*-Value	F-Value	*p*-Value
Material, $X_{0}$	2	5696.57	0.00	754.11	0.00	363.77	0.00	65.02	0.000
Infill density, $X_{1}$	2	3641.76	0.00	3567.47	0.00	272.80	0.00	16.85	0.000
Printing orientation, $X_{2}$	2	1077.96	0.00	596.24	0.00	27.14	0.00	4.48	0.014
$X_{0}\cdot X_{1}$	4	42.67	0.00	15.51	0.00	10.58	0.00	11.62	0.000
$X_{0}\cdot X_{2}$	4	165.29	0.00	30.47	0.00	15.01	0.00	2.74	0.033
$X_{1}\cdot X_{2}$	4	85.84	0.00	236.55	0.00	26.08	0.00	3.80	0.006
$X_{0}\cdot X_{1}\cdot X_{2}$	8	33.20	0.00	10.91	0.00	2.67	0.01	4.22	0.000
Error	108								
Total	134								

**Table 5:** Summary of the regression models for 3D-printed materials.

Material	Regression Model	R
PA	$E\;(\mathrm{MPa})=617.4+9.112\;X_{1}$ $-10.181\;X_{2}$ $+0.1118\;X_{1}$ $X_{2}$	0.99
	$TS\;(\mathrm{MPa})=17.53+0.221\;X_{1}$ $-0.3044\;X_{2}$ $+0.0034\;X_{1}$ $X_{2}$	0.97
CF/PA	$E\;(\mathrm{MPa})=1075+18.84\;X_{1}$ $-\;4.42\;X_{2}$ $-0.0432\;X_{1}$ $X_{2}$	0.93
	$TS\;(\mathrm{MPa})=23.53+0.2925\;X_{1}$ $-0.2603\;X_{2}$ $+0.0019\;X_{1}$ $X_{2}$	0.94
GF/PA	$E\;(\mathrm{MPa})=770.6+11.914\;X_{1}$ $-8.16\;X_{2}$ $+0.0556\;X_{1}$ $X_{2}$	0.98
	$TS\;(\mathrm{MPa})=18.71+0.2671\;X_{1}$ $-0.2713\;X_{2}$ $+0.0024\;X_{1}$ $X_{2}$	0.98

**Table 6:** Variation of R and MSE with the number of neurons in the hidden layer for the ANN with one output.

Material	Output Variable	ANN Model	R	MSE
PA	E	2-1-1	0.80	3.43 × 10^4^
		2-2-1	1.00	5.93 × 10^2^
		2-3-1	1.00	5.99 × 10^−23^
	TS	2-1-1	0.95	6.66 × 10^0^
		2-2-1	0.99	1.87 × 10^0^
		2-3-1	1.00	3.94 × 10^−28^
CF/PA	E	2-1-1	0.94	2.47 × 10^4^
		2-2-1	1.00	1.68 × 10^3^
		2-3-1	1.00	4.67 × 10^−1^
	TS	2-1-1	0.95	8.51 × 10^0^
		2-2-1	0.94	1.09 × 10^1^
		2-3-1	1.00	8.14 × 10^−24^
GF/PA	E	2-1-1	0.98	4.60 × 10^3^
		2-2-1	0.98	4.52 × 10^3^
		2-3-1	1.00	2.77 × 10^−23^
	TS	2-1-1	0.97	5.24 × 10^0^
		2-2-1	0.99	1.22 × 10^0^
		2-3-1	1.00	9.96 × 10^−19^

**Table 7:** Weights and biases for the ANN model for Young’s modulus of 3D-printed samples.

	PA (2-3-1)			GF/PA (2-3-1)			CF/PA (2-3-1)		
$W_{i,j}$	The input-to-hidden layer weights								
1	0.862	0.881		2.078	2.737		−15.823	30.610	
2	4.284	−0.373		1.819	0.158		17.500	−2.182	
3	−3.818	−0.567		−0.711	−2.069		1.006	−15.697	
$W_{j,k}$	The hidden-to-output layer weights								
	0.404	1.132	1.727	−0.231	0.940	0.789	−0.213	0.356	1.318
$b_{1}$	The input-to-hidden layer biases								
1	0.786			−0.873			−14.671		
2	0.506			0.073			15.477		
3	−4.278			−2.758			−17.064		
$b_{2}$	The hidden-to-output layer bias								
	1.197			0.605			0.887		

**Table 8:** Weights and biases for the ANN model for tensile strength of 3D-printed samples.

	PA (2-3-1)			GF/PA (2-3-1)			CF/PA (2-3-1)		
$W_{i,j}$	The input-to-hidden layer weights								
1	1.742	2.643		0.354	3.454		2.855	6.158	
2	0.456	−1.740		0.288	−1.793		0.617	0.195	
3	−2.301	−3.341		−2.972	−5.855		−2.433	−1.894	
$W_{j,k}$	The hidden-to-output layer weights								
	0.877	1.053	−0.434	2.945	4.199	−0.504	−0.412	2.509	1.600
$b_{1}$	The input-to-hidden layer biases								
1	−2.560			−1.885			−2.542		
2	0.521			0.974			0.715		
3	−1.838			−3.808			−4.440		
$b_{2}$	The hidden-to-output layer bias								
	0.366			−0.602			0.147		

**Table 9:** Pearson’s correlation for testing of ANN model with a single output.

Material	Young’s Modulus		Tensile Strength	
	*R*	*p*-Value	*R*	*p*-Value
PA	0.997	0.000	0.992	0.000
CF/PA	0.999	0.000	0.997	0.000
GF/PA	0.996	0.000	0.998	0.000

**Table 10:** Variation of R and MSE with the number of neurons in the hidden layer for the ANN with two outputs.

Material	ANN Model	R	MSE
PA	2-1-2	0.996	2.97 × 10^3^
	2-2-2	1.000	2.98 × 10^2^
	2-3-2	1.000	3.46 × 10^−1^
GF/PA	2-1-2	0.998	2.30 × 10^3^
	2-2-2	1.000	5.06 × 10^2^
	2-3-2	1.000	9.54 × 10^−1^
CF/PA	2-1-2	0.99	1.24 × 10^4^
	2-2-2	1.00	8.42 × 10^2^
	2-3-2	1.00	1.94 × 10^3^
	2-4-2	1.00	2.26 × 10^−1^

**Table 11:** Weights and biases for the ANN model with two outputs (e.g., Young’s modulus and tensile strength) of 3D-printed samples.

	GF/PA (2-3-2)			CF/PA (2-4-2)				PA (2-3-2)		
$w_{i,j}$	The input-to-hidden layer weights									
1	8.410	3.722		5.122	1.020			−3.116	−0.888	
2	1.377	−0.051		13.399	−4.141			4.560	−3.978	
3	0.962	0.911		0.344	2.500			−5.075	−0.745	
4				2.613	1.336					
$w_{j,k}$	The hidden-to-output layer weights									
1	−0.481	1.435	−1.542	1.749	−1.634	−0.888	0.796	−0.390	0.568	−0.153
2	−0.280	1.277	−1.612	4.422	−4.134	−1.106	0.942	−0.371	0.586	−0.102
$b_{1}$	The input-to-hidden layer biases									
1	−4.582			−3.341				1.483		
2	−0.190			−5.134				4.259		
3	2.265			0.408				−4.758		
4				1.411						
$b_{2}$	The hidden-to-output layer biases									
1	1.351			−0.113				−0.105		
2	1.565			0.321				−0.074		

**Table 12:** Pearson’s correlation for training and testing of ANN model with two outputs.

Material	Training				Testing			
	Elastic Modulus		Tensile Strength		Elastic Modulus		Tensile Strength	
	*R*	*p*-Value	*R*	*p*-Value	*R*	*p*-Value	*R*	*p*-Value
PA	1.00	0.000	0.995	0.000	0.997	0.000	0.996	0.000
CF/PA	1.00	0.000	0.997	0.000	0.999	0.000	0.995	0.000
GF/PA	1.00	0.000	0.987	0.000	0.996	0.000	0.984	0.000

**Table 13:** Pearson’s correlation for ANN models considering the entire data set.

Material	ANN Model with One Output				ANN Model with Two Outputs			
	Young’s Modulus		Tensile Strength		Young’s		Tensile Strength	
	*R*	*p*-Value	*R*	*p*-Value	*R*	*p*-Value	*R*	*p*-Value
PA	0.998	0.000	0.996	0.000	0.998	0.000	0.996	0.000
CF/PA	0.999	0.000	0.999	0.000	0.999	0.000	0.996	0.000
GF/PA	0.998	0.000	0.999	0.000	0.998	0.000	0.985	0.000

**Table 14:** Results for the two-sample *t* test.

Material	ANN Model with One Output				ANN Model with Two Outputs			
	Young’s Modulus		Tensile Strength		Young’s Modulus		Tensile Strength	
	*T*-Value	*p*-Value	*T*-Value	*p*-Value	*T*-Value	*p*-Value	*T*-Value	*p*-Value
PA	−0.01	0.993	−0.02	0.986	−0.01	0.993	−0.02	0.984
CF/PA	−0.05	0.965	0.03	0.978	−0.05	0.965	0.03	0.976
GF/PA	0.02	0.984	−0.03	0.989	0.02	0.984	−0.03	0.980

**Table 15:** Correlation between ANN and regression modeling.

Material	ANN Model with One Output				ANN Model with Two Outputs			
	Young’s Modulus		Tensile Strength		Young’s Modulus		Tensile Strength	
	*R*	*p*-Value	*R*	*p*-Value	*R*	*p*-Value	*R*	*p*-Value
PA	0.958	0.000	0.962	0.000	0.938	0.000	0.946	0.000
GF/PA	0.975	0.000	0.940	0.000	0.949	0.000	0.977	0.000
CF/PA	0.808	0.000	0.924	0.989	0.889	0.000	0.836	0.000

## Data Availability

The original contributions presented in this study are included in the article. Further inquiries can be directed to the corresponding author.

## Appendix A

**Table A1:** ANOVA for Young’s modulus (*p*-value < 0.05).

Source	DF	Adj SS	Adj MS	F-Value	*p*-Value
Material, $X_{0}$	2	20,388,272	10,194,136	5696.57	0.00
Infill density, $X_{1}$	2	13,034,036	6,517,018	3641.76	0.00
Printing orientation, $X_{2}$	2	3,858,066	1,929,033	1077.96	0.00
$X_{0}\cdot X_{1}$	4	305,461	76,365	42.67	0.00
$X_{0}\cdot X_{2}$	4	1,183,152	295,788	165.29	0.00
$X_{1}\cdot X_{2}$	4	614,470	153,617	85.84	0.00
$X_{0}\cdot X_{1}\cdot X_{2}$	8	475,354	59,419	33.20	0.00
Error	108	193,268	1790		
Total	134	40,052,078			

**Table A2:** ANOVA for tensile strength (*p*-value < 0.05).

Source	DF	Adj SS	Adj MS	F-Value	*p*-Value
Material, $X_{0}$	2	1684.0	842.02	754.11	0.00
Infill density, $X_{1}$	2	7966.7	3983.33	3567.47	0.00
Printing orientation, $X_{2}$	2	1331.5	665.74	596.24	0.00
$X_{0}\cdot X_{1}$	4	69.3	17.31	15.51	0.00
$X_{0}\cdot X_{2}$	4	136.1	34.02	30.47	0.00
$X_{1}\cdot X_{2}$	4	1056.5	264.13	236.55	0.00
$X_{0}\cdot X_{1}\cdot X_{2}$	8	97.5	12.18	10.91	0.00
Error	108	120.6	1.12		
Total	134	12,462.1			

**Table A3:** ANOVA for stress at break (*p*-value < 0.05).

Source	DF	Adj SS	Adj MS	F-Value	*p*-Value
Material, $X_{0}$	2	6521.8	3260.89	363.77	0.00
Infill density, $X_{1}$	2	4890.8	2445.40	272.80	0.00
Printing orientation, $X_{2}$	2	486.6	243.28	27.14	0.00
$X_{0}\cdot X_{1}$	4	379.3	94.83	10.58	0.00
$X_{0}\cdot X_{2}$	4	538.1	134.53	15.01	0.00
$X_{1}\cdot X_{2}$	4	935.1	233.78	26.08	0.00
$X_{0}\cdot X_{1}\cdot X_{2}$	8	191.8	23.97	2.67	0.01
Error	108	968.1	8.96		
Total	134	14,911.6			

**Table A4:** ANOVA for strain at break (*p*-value < 0.05).

Source	DF	Adj SS	Adj MS	F-Value	*p*-Value
Material, $X_{0}$	2	1.1456	0.5728	65.02	0.000
Infill density, $X_{1}$	2	0.2968	0.1484	16.85	0.000
Printing orientation, $X_{2}$	2	0.0789	0.0394	4.48	0.014
$X_{0}\cdot X_{1}$	4	0.4095	0.1024	11.62	0.000
$X_{0}\cdot X_{2}$	4	0.0964	0.0241	2.74	0.033
$X_{1}\cdot X_{2}$	4	0.1340	0.0335	3.80	0.006
$X_{0}\cdot X_{1}\cdot X_{2}$	8	0.2974	0.0372	4.22	0.000
Error	108	0.9514	0.0088		
Total	134	3.4098			

## Appendix B

**Table A5:** Testing data for 3D-printed PA using the ANN model (2-3-1) with one output.

Infill Density (%)	Printing Orientation (°)	$E_{EXP}$ (MPa)	$E_{ANN}$ (MPa)	ε (%)	${TS}_{EXP}$ (MPa)	${TS}_{ANN}$ (MPa)	ε (%)
50	0	1126.00	1116.70	−0.83	30.56	30.04	−1.71
50	45	768.54	812.40	5.71	20.76	21.26	2.45
50	90	675.51	677.40	0.28	16.53	16.51	−0.13
75	0	1267.90	1246.60	−1.68	31.58	30.98	−1.90
75	45	1210.90	1261.00	4.14	31.85	34.72	9.00
75	90	1152.50	1137.10	−1.34	30.43	29.24	−3.91
100	0	1579.20	1537.10	−2.67	40.42	39.94	−1.17
100	45	1596.20	1589.90	−0.39	42.35	42.24	−0.26
100	90	1589.50	1600.80	0.71	41.45	41.67	0.53

**Table A6:** Testing data for 3D-printed GF/PA using the ANN model (2-3-1) with one output.

Infill Density (%)	Printing Orientation (°)	$E_{EXP}$ (MPa)	$E_{ANN}$ (MPa)	ε (%)	${TS}_{EXP}$ (MPa)	${TS}_{ANN}$ (MPa)	ε (%)
50	0	1402.30	1439.60	2.66	33.79	34.21	1.24
50	45	956.68	1011.90	5.77	22.63	24.09	6.44
50	90	905.28	907.80	0.28	19.05	19.00	−0.26
75	0	1649.10	1624.30	−1.50	34.62	34.80	0.51
75	45	1552.60	1495.10	−3.70	36.99	36.33	−1.79
75	90	1387.30	1359.30	−2.02	28.90	29.78	3.03
100	0	2022.10	2023.00	0.04	47.71	46.94	−1.62
100	45	1782.90	1757.10	−1.45	44.74	44.09	−1.44
100	90	1732.90	1741.40	0.49	42.32	42.54	0.50

**Table A7:** Testing data for 3D-printed CF/PA using the ANN model (2-3-1) with one output.

Infill Density (%)	Printing Orientation (°)	$E_{EXP}$ (MPa)	$E_{ANN}$ (MPa)	ε (%)	${TS}_{EXP}$ (MPa)	${TS}_{ANN}$ (MPa)	ε (%)
50	0	2090.00	2112.20	1.06	41.47	40.70	−1.86
50	45	1453.90	1455.30	−0.10	26.75	26.89	0.54
50	90	1362.30	1414.10	−3.80	23.36	22.76	−2.58
75	0	2482.30	2467.60	0.59	41.10	41.32	0.52
75	45	2353.50	2337.10	0.70	47.86	46.30	−3.24
75	90	1923.50	1991.80	−3.55	36.94	37.64	1.91
100	0	3078.10	3036.80	1.34	53.87	54.26	0.73
100	45	2336.50	2337.10	−0.03	48.22	48.03	−0.39
100	90	2118.40	2144.50	−1.23	44.64	45.09	1.03

## Appendix C

**Table A8:** Testing data for the 3D-printed PA using the ANN model (2-3-2) with two outputs.

Infill Density (%)	Printing Orientation (°)	$E_{EXP}$ (MPa)	$E_{ANN}$ (MPa)	ε (%)	${TS}_{EXP}$ (MPa)	${TS}_{ANN}$ (MPa)	ε (%)
50	0	1126.00	1116.70	−0.83	30.56	30.20	−1.19
50	45	768.54	812.40	5.71	20.76	21.00	1.17
50	90	675.51	677.40	0.28	16.53	16.60	0.40
75	0	1267.90	1246.60	−1.68	31.58	32.60	3.22
75	45	1210.90	1261.00	4.14	31.85	33.00	3.60
75	90	1152.50	1137.10	−1.34	30.43	29.30	−3.72
100	0	1579.20	1537.10	−2.67	40.42	40.30	−0.29
100	45	1596.20	1589.90	−0.39	42.35	41.70	−1.53
100	90	1589.50	1600.80	0.71	41.45	42.00	1.32

**Table A9:** Testing data for 3D-printed GF/PA using the ANN model (2-3-2) with two outputs.

Infill Density (%)	Printing Orientation (°)	$E_{EXP}$ (MPa)	$E_{ANN}$ (MPa)	ε (%)	${TS}_{EXP}$ (MPa)	${TS}_{ANN}$ (MPa)	ε (%)
50	0	1402.30	1439.60	2.66	33.79	34.10	0.91
50	45	956.68	1011.90	5.77	22.63	23.00	1.64
50	90	905.28	907.80	0.28	19.05	20.30	6.56
75	0	1649.10	1624.30	−1.51	34.62	36.60	5.72
75	45	1552.60	1495.10	−3.70	36.99	33.30	−9.98
75	90	1387.30	1359.30	−2.02	28.90	30.90	6.92
100	0	2022.10	2023.00	0.04	47.71	47.40	−0.65
100	45	1782.90	1757.10	−1.45	44.74	43.30	−3.21
100	90	1732.90	1741.40	0.48	42.32	42.90	1.36

**Table A10:** Testing data for 3D-printed CF/PA using the ANN model (2-4-2) with two outputs.

Infill Density (%)	Printing Orientation (°)	$E_{EXP}$ (MPa)	$E_{ANN}$ (MPa)	ε (%)	${TS}_{EXP}$ (MPa)	${TS}_{ANN}$ (MPa)	ε (%)
50	0	2090.00	2112.20	1.06	41.47	41.50	0.06
50	45	1453.90	1455.30	0.10	26.75	25.50	−4.67
50	90	1362.30	1414.10	3.80	23.36	23.80	1.88
75	0	2482.30	2467.60	−0.59	41.10	41.30	0.47
75	45	2353.50	2284.20	−0.63	47.86	45.60	−4.71
75	90	1923.50	1986.70	3.55	36.94	37.80	2.33
100	0	3078.10	3036.80	−1.34	53.87	54.30	0.80
100	45	2336.50	2335.70	−0.03	48.22	48.00	−0.45
100	90	2118.40	2144.50	1.23	44.64	45.10	1.04
